# PERCEPTIONS OF DEMENTIA

**DOI:** 10.1093/geroni/igz038.2779

**Published:** 2019-11-08

**Authors:** Paul Kingston, Louise Taylor, Charlotte Eost-Telling, and Jan Bailey

**Affiliations:** University of Chester, Chester, England, United Kingdom

## Abstract

This paper considers narratives of 143 respondents (“Observers”) to a Mass Observation Project Directive exploring individuals’ perceptions of dementia. Perceptions of dementia held by “Observers” with experience of dementia and those without differed sharply. “Observers” with experience of dementia offered insight into living with and caring for a person with dementia, and the impact this had on their lives and personal relationships. Whereas, “Observers” with no direct experience of dementia focused more on common disease symptoms such as memory loss and reflected idealised views of care. “Observers” often feared being diagnosed with dementia themselves. This suggests education to facilitate care planning and ameliorate fears held by the public is required.

